# ﻿Studies on *Typhonium* (Araceae) of Thailand I: *Typhoniumvinicolor*, a new species from Khon Kaen Province, Northeastern Thailand

**DOI:** 10.3897/phytokeys.246.128778

**Published:** 2024-09-03

**Authors:** Piyaporn Saensouk, Surapon Saensouk, Khant Zaw Hein, Thawatphong Boonma, Anousone Sengthong, Sarayut Rakarcha

**Affiliations:** 1 Diversity of Family Zingiberaceae and Vascular Plant for Its Applications Research Unit, Department of Biology, Faculty of Science, Mahasarakham University, Kantarawichai District, Maha Sarakham 44150, Thailand; 2 Diversity of Family Zingiberaceae and Vascular Plant for Its Applications Research Unit, Walai Rukhavej Botanical Research Institute, Mahasarakham University, Kantarawichai District, Maha Sarakham 44150, Thailand; 3 Ta Yote Tan Street, Monywa, 02301, Sagaing Region, Myanmar; 4 Brio Botanical Research Garden, 53 M. 5 Phikun-ok, Ban Na District, Nakhon Nayok 26110, Thailand; 5 Faculty of Forest Science, National University of Laos, Vientiane Capital, 7322, Laos; 6 Queen Sirikit Botanic Garden, The Botanical Garden Organization, Chiang Mai 50180, Thailand

**Keywords:** Areae, Aroideae, Indochina, Northeastern Thailand, plant taxonomy

## Abstract

*Typhoniumvinicolor* from Khon Kaen Province (Northeastern Thailand), is described and illustrated as a species new to science. Color plates, phenology, distribution, discussion of similar taxa, and conservation status assessment are provided.

## ﻿Introduction

*Typhonium*Schott (1829) (Araceae Juss.) is a genus of tuberous (sometimes rhizomatous or stoloniferous), terrestrial, and seasonally dormant herbs that inhabit forest floors, rocky areas, wet sites, stream sides, and grassy places in tropical and subtropical humid and seasonal forests, as well as in agricultural land (Mayo et al. 1997; Low et al. 2020). Hay et al. (2022) transferred most of the Australian *Typhonium* species to *Lazarum* A.Hay, so, according to these authors *Typhonium* (*sensu stricto*) would refer to species mainly distributed in Indochina (see e.g., Low et al. 2020; Hay and Hein 2022). Among the genera of tribe Areae, *Typhoniums.str.* is the largest one, with more than 70 species (100 based on Boyce and Croat 2011; 72 in POWO 2024). The highest species diversity of *Typhonium* is found in Thailand, with 32 species, 24 of which are endemic (Boyce et al. 2012). Later than Boyce et al. (2012), six new species have been described from Thailand (Galloway 2012, 2015; Hetterscheid 2013; Sookchaloem and Maneeanakekul 2018), increasing the total number of species in the country to 38. However, the current number of species occurring in Thailand is undoubtedly much higher (Boyce et al. 2012).

During our botanical survey in Khon Kaen Province (Northeastern Thailand), an unknown species of *Typhonium* was collected by the second author (SS). After meticulously examining its morphology and comparing it with protologues and relevant literature, as well as with digitized type specimens from Thailand and neighboring countries, it became apparent that the collected specimen does not match any other known *Typhonium* species. Thus, we consider it to represent a taxonomic novelty, which is described and illustrated in the present paper.

## ﻿Materials and methods

The measurements and descriptions were based on freshly collected and/or alcohol-preserved material, processed according to the methods established by Davies et al. (2023). The species description follows Hay and Hein (2022), while Araceae morphological terminology follows Mayo et al. (1997) implemented by the descriptive terminology of Beentje (2016). Relevant type specimens of *Typhonium* species from Thailand and neighboring countries were examined in different herbaria (A, AAU, B, BK, BKF, C, CAL, CMU, E, HITBC, K, KKU, KUN, L, M, MO, P, PE, QBG, SING, and WAG; acronyms follow Thiers 2024) through high-resolution images from https://plants.jstor.org/ and Global Biodiversity Information Facility (GBIF) accessed from https://www.gbif.org. An assessment of conservation status was carried out following IUCN (2024), based on our current knowledge and the respective terminology on categories, criteria, and subcriteria. The photographs in the plate were taken with an iPhone 13 (iOS version 17.5.1, 2021, Apple Inc., Cupertino, CA, USA). The figure in this study was created using Pixelmator Pro (Version 3.6.5, Archipelago, 2023, Pixelmator Team, Vilnius, Lithuania) on a MacBook Pro (13-inch, M1, 2020, Apple Inc., Cupertino, CA, USA).

## ﻿Taxonomic treatment

### ﻿ Typhonium vinicolor

Taxon classificationPlantaeAlismatalesAraceae

P.Saensouk, K.Z.Hein & Saensouk sp. nov.

F11E32F7-7B4C-58DE-9C5D-E0728A1BF694

urn:lsid:ipni.org:names:77347851-1

Fig. 1

#### Type.

Thailand • Northeastern – Khon Kaen Province, 13 May 2023, *Surapon Ara001* (holotype KKU!; isotypes FOF!, MSU!).

**Figure 1.**
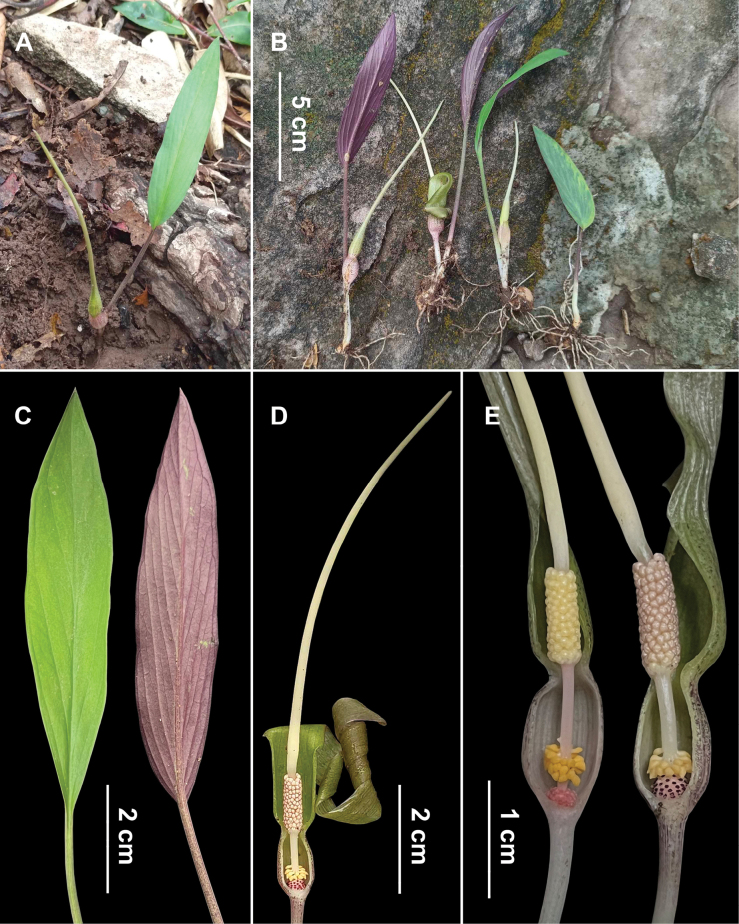
*Typhoniumvinicolor***A** plant in habitat **B** excavated flowering plants **C** leaf blade (left showing adaxial surface, right showing abaxial surface) **D** inflorescence at pistillate anthesis, nearside spathe artificially removed **E** spadix at pistillate anthesis, nearside spathe artificially removed. Photos by: Surapon Saensouk and Thawatphong Boonma.

#### Diagnosis.

*Typhoniumvinicolor* is easily distinguishable from the other *Typhonium* species by having narrowly elliptic to elliptic-lanceolate leaf blades with a reddish-purple abaxial surface. An only exception is *T.laoticum* Gagnep. (Gagnepain 1942), which shows similar leaf blades. However, *T.vinicolor* differs from *T.laoticum* by its reddish-purple abaxial surface of leaf blades (*vs.* pale green), white or pale green spathe with dark purple mottling externally (*vs.* pink spathe with brown mottling externally), pistillate zone with 5–6 pistil rows (*vs.* 2–3 pistil rows), and staminodes more or less loosely arranged in 4–5 spirals (*vs.* staminodes densely arranged in 2–3 spirals).

#### Description.

Small, deciduous, herbs, to 15 cm tall. ***Stem*** hypogeal, subglobose or depressed globose tuber, 1.2–1.5 cm in diameter, externally pale brown, internally white. ***Roots*** filiform, 0.6–1.0 mm in diameter, white. ***Leaves*** 1–2(–3) together; ***petioles*** 6.5–8.5 cm long, ca. 0.2 cm in diameter, erect, slender, terete, glabrous, basal subterranean portion white, upper aerial portion pale green with numerous longitudinal dark purple striations and spots; ***petiolar sheath*** 2.5–3.0 cm long, ca. 1/3 of petiole length; ***leaf blade*** 8.0–13.0 × 1.8–4.2 cm, narrowly elliptic to elliptic-lanceolate, or elliptic-oblanceolate, chartaceous, adaxially medium green, abaxially reddish-purple, glabrous on both sides, base cuneate or obtuse, margin entire, apex acute and mucronate, mucro ca. 1 mm long; ***midrib*** adaxially impressed, abaxially raised, rounded, 1.5–1.8 mm wide at the base, ca. 1.0 mm wide at center, then narrowing towards blade apex; ***primary lateral veins*** 5–7 per side, adaxially impressed, abaxially raised, diverging from the midrib at 15–30°, anastomosing at 0.5–1.5 mm from margin into an intramarginal collective vein; ***higher order venation*** reticulate. ***Inflorescence*** solitary, subtended by a cataphyll; ***cataphyll*** up to 3.0 cm long, linear-lanceolate, membranous, semi-hyaline, greenish white or white, later withering brown; ***peduncle*** 2.8–3.2 cm long, ca. 0.2 cm in diameter, almost entirely subterranean, white, terete, glabrous; ***spathe*** 8.5–9.5 cm long, strongly differentiated into a spathe tube and a spathe limb by a constriction; ***spathe tube*** ca. 1.2 cm long, 0.6–0.7 cm in diameter, convolute, ellipsoid-ovoid, externally white or greenish white with a dense dark purple mottling, internally greenish white; ***spathe limb*** 7.3–8.3 cm long, 0.6–0.7 cm in diameter at base, linear-lanceolate, tapering towards apex, externally green or yellowish-green with dark purple mottling, internally pale yellowish green, basal part of limb shortly convolute and erect, upper part reflexed and then strongly coiled at anthesis, apex narrowly acute. ***Spadix*** sessile, 8.0–9.0 cm long, nearly as long as or shorter than spathe; ***pistillate zone*** ca. 2 mm long, ca. 3 mm in diameter at the base, shortly conical, with 5–6 rows of congested pistils; ***ovary*** ca. 0.7 mm high, ca. 0.5 mm in diameter, obovoid, white, unilocular with one basal ovule held obliquely on a funicle, on a basal placenta; ***style*** very short, ca. 0.1 mm high, ca. 2.5 mm in diameter; ***stigma*** ca. 0.3 mm in diameter, discoid, red, papillate; ***sterile interstice*** between pistillate and staminate zones 0.8–1.0 cm long, ca. 0.1 cm in diameter, upper part naked, terete, glabrous, glossy white, lower ca. 0.2 cm covered with 4–5 spirals of staminodes; ***staminodes*** 1.2–1.5 mm long, 0.5–0.7 mm in diameter at widest point, clavate-fusiform, shortly beaked with acute apex, free, slightly distant from each other, perpendicular to the spadix axis or slightly curved downwards, glabrous, yellow; ***staminate zone*** 0.7–1.0 cm long, ca. 0.3 cm in diameter, cylindric; ***stamens*** congested, not ostensibly arranged into staminate flowers, irregularly 4-lobed, 0.6–0.7 mm in diameter, pink or yellow, dehiscing by an apical pore; ***appendix*** sessile, 6.2–7.0 cm long, 1.5–2.0 mm in diameter, narrowly elongate-conical, tapering towards apex, erect or weakly arching, glabrous, ivory-colored, base slightly attenuate, apex acute. ***Infructescence*** not seen.

#### Etymology.

The specific epithet is derived from the Latin “*vinicolor*” (wine-coloured), referring to the reddish-purple abaxial surface of the leaves.

#### Proposed vernacular name.

Uttapit-See-Wine.

#### Phenology.

Flowering time in May.

#### Distribution and habitat.

The newly discovered species is found exclusively at its type locality in Khon Kaen Province (Northeastern Thailand). It thrives in shaded to semi-shaded areas of tropical deciduous forests at elevations ranging from 200 to 250 m a.s.l. The species shows optimal growth in sandy loam soil mixed with rocks.

#### Conservation status.

This new species is known exclusively from its type locality, and no sufficient information there is regarding potential threats to its habitat. In accordance with the Red List criteria of the IUCN Standards and Petitions Subcommittee (2024), we propose classifying this species as ‘Data Deficient’ (DD). Further research is necessary, as there is inadequate information to assess the conservation status of this species. Currently, data is limited regarding its distribution, with no details on population size, trends, or potential threats to its habitat.

#### Taxonomic notes.

Based on overall morphology, this new species is also similar to *Typhoniumgriseum* Hett. & Sookch. (Hetterscheid et al. 2001), which is a sister species of *T.laoticum* (Low et al. 2020). But *T.vinicolor* is strikingly different from *T.griseum* in having the leaf blade base cuneate or obtuse (vs. with rounded posterior lobes), spadix nearly as long as, or shorter than, spathe (vs. longer than spathe), clavate-fusiform and yellow staminodes (vs. narrowly fusiform, sickle-shaped, white staminodes), longer staminate zone (ca. 1 cm vs. ca. 0.5 cm long), and erect or weakly arching, ivory-colored appendix (vs. strongly arching, pale brown appendix).

Regarding spathe and spadix structures, the new species also resembles *Typhoniumhuense* V.D.Nguyen & Croat (Nguyen and Croat 1997), *T.lineare* Hett. & V.D.Nguyen (Hetterscheid and Nguyen 2001) and *T.stigmatilobatum* V.D.Nguyen (Nguyen 2008) from Vietnam. However, the latter three species differ from *T.vinicolor* by having horizontally flexed spathe limb at anthesis (vs. only the upper part of spathe limb reflexed and then strongly coiled at anthesis in *T.vinicolor*) and strongly arching, dark brown or violet, stipitate appendix (vs. erect or weakly arching, ivory, sessile appendix in *T.vinicolor*). A more detailed comparison between *T.vinicolor* and its morphologically allied species is presented in Table 1.

**Table 1.** Morphological comparison of *Typhoniumvinicolor* and its allied species, *T.griseum* (Hetterscheid et al. 2001), *T.huense* (Nguyen and Croat 1997), *T.laoticum* (Gagnepain 1942; Boyce et al. 2012), *T.lineare* (Hetterscheid and Nguyen 2001) and *T.stigmatilobatum* (Nguyen 2008).

	*T.griseum*	*T.huense*	*T.laoticum*	*T.lineare*	*T.stigmatilobatum*	*T.vinicolor*
**Leaf blade**	orbicular, triangular cordate or narrowly ovate	triangular-cordate to deeply trilobed	lanceolate or elliptic-oblong	pedatisect with linear or linear-lanceolate lobes	deeply trilobed or pedatisect with ovate to oblong-ovate lobes	narrowly elliptic to elliptic-lanceolate
**Spathe tube**	ca. 1 cm long, externally dirty white with brownish-red spots	ca. 1.5 cm long, externally pale green with purplish violet spots	1.3–1.5 cm long, externally pink with brown striations and spots	up to 1.8 cm long, externally whitish-gray with blackish-gray striations and spots	ca. 1.3 cm long, externally pale green or dull white with black or brown spots	ca. 1.2 cm long, externally white or greenish-white with a dense dark purple mottling
**Spathe limb**	ca. 10 times longer than spathe tube	ca. 6 times longer than spathe tube	4–5 times longer than spathe tube	7–8 times longer than spathe tube	10–12 times longer than spathe tube	6–7 times longer than spathe tube
**Spadix**	longer than spathe, ca. 13 cm long	as long as or slightly longer than spathe, up to 11 cm long	shorter than the spathe, ca. 7 cm long	as long as or slightly shorter than spathe, up to 17 cm long	as long as spathe, up to 17 cm long	nearly as long as or shorter than spathe, 8.0–9.0 cm long
**Staminodes**	narrowly fusiform, sickle-shaped, curved downwards, white	fusiform, variously directed, yellowish white	clavate, perpendicular to the spadix axis or curved upwards, white	subulate or fusiform, variously directed, white or yellow	fusiform, perpendicular to the spadix axis or slightly curved downwards, dull white	clavate-fusiform, perpendicular to the spadix axis or slightly curved downwards, yellow
**Appendix**	sessile, ca. 12 cm long, strongly arching, pale brown	stipitate, ca. 9 cm long, strongly arching, brown or violet	sessile, ca. 5 cm long, erect, pale brown	stipitate, ca. 15 cm long, strongly arching, brown or golden yellow	sessile, 14–15 cm long, strongly arching, dark brown	sessile, 6.2–7.0 cm long, erect or weakly arching, ivory-colored

#### Additional specimens examined (paratypes).

Thailand • Northeastern – Khon Kaen Province, 18 May 2024, *Saensouk, Boonma & Sengthong, SS 24518* (FOF!).

## Supplementary Material

XML Treatment for Typhonium vinicolor

## ﻿Additional information

### Author ORCIDs

Surapon Saensouk https://orcid.org/0000-0002-9178-188X

Khant Zaw Hein https://orcid.org/0000-0002-8735-9264

Thawatphong Boonma https://orcid.org/0000-0003-2324-4967

### Data availability

All of the data that support the findings of this study are available in the main text.
